# Size of Subcortical Band Heterotopia Influences the Susceptibility to Hyperthermia-Induced Seizures in a Rat Model

**DOI:** 10.3389/fncel.2019.00473

**Published:** 2019-10-18

**Authors:** Kalliopi Moustaki, Emmanuelle Buhler, Robert Martinez, Françoise Watrin, Alfonso Represa, Jean-Bernard Manent

**Affiliations:** Institut de Neurobiologie de la Méditerranée INMED, INSERM UMR 1249, Aix-Marseille University, Marseille, France

**Keywords:** malformation of cortical development, gray matter heterotopia, febrile seizure, hyperthermic seizures, early life seizures

## Abstract

Studies conducted in human and rodent models have suggested that preexisting neurodevelopmental defects could predispose immature brains to febrile seizures (FS). However, the impact of the anatomical extent of preexisting cortical malformations on FS susceptibility was never assessed. Here, we induced hyperthermic seizures (HS) in rats with bilateral subcortical band heterotopia (SBH) and found variable degrees of HS susceptibility depending on inter-individual anatomical differences in size and extent of SBH. This indicates that an association exists between the overall extent or location of a cortical malformation, and the predisposition to FS. This also suggests that various predisposing factors and underlying causes may contribute to the etiology of complex FS.

## Introduction

Febrile seizures (FS) are the most common seizure disorder in childhood, affecting 2–5% of children (Nelson and Ellenberg, 1978; Berg et al., 1992). Simple FS is defined as a short generalized seizure, of a duration of <15 min, not recurring within 24 h, occurring during a febrile episode not caused by an acute disease of the nervous system, in a child aged 6 months to 5 years, with no neurologic deficits. Complex FS is defined as a focal, or generalized and prolonged seizure, of a duration of >15 min, recurring more than once in 24 h, and/or associated with postictal neurologic abnormalities, more frequently a postictal palsy, or with previous neurologic deficits [ILAE taskforce (Capovilla et al., 2009)].

It has long been known that children with a history of ante- and perinatal genetic or acquired injuries are more likely to develop FS, suggesting that preexisting developmental defects could predispose immature brains to FS, although neuroradiological evidences were initially lacking (Berg and Shinnar, 1996; Wallace, 1972). These evidences were later obtained, revealing a high incidence of MRI-detected brain abnormalities in patient cohorts with both simple and complex FS (Hesdorffer et al., 2008). Among identified abnormalities, malformations of cortical development (MCDs) such as focal cortical dysplasia were detected (Hesdorffer et al., 2008), sometime co-occurring with other defects (Barba et al., 2014). Various incidences of FS were also described in patient cohorts or case reports for other types of MCDs, such as periventricular nodular heterotopia (Fallil et al., 2015) or subcortical band heterotopia (SBH) (Barkovich et al., 1994; Bahi-Buisson et al., 2013). Last, subtle preexisting hippocampal malformations have been suggested to favor FS and contribute to temporal lobe epilepsy (Fernandez et al., 1998).

Rodent models of hyperthermia-induced FS (referred to as hyperthermic seizures or HS in the present report) have been developed [reviewed in Koyama and Matsuki (2010)], and the susceptibility to HS was tested in models of cortical dysplasia and MCDs. In P14 rat pups with cortical dysplasia induced by prenatal exposure to methylazoxymethanol (MAM), HS were found to occur more frequently than in control littermates, and with a higher mortality rate (Germano et al., 1996). This observation was however not replicated in P10 rats, where similar temperature thresholds were found (Park et al., 2010). In P10 rats with localized microgyri induced by cortical freezing, HS were found to occur with shorter latencies and lower temperature thresholds than in non-lesioned littermates (Scantlebury et al., 2004).

In all, human and rodent studies suggest that MCDs in immature brains may predispose to FS and/or affect temperature threshold and latencies for seizures. However, whether an association exists between the size of MCDs and the predisposition to FS remains an open question. Here, we examined whether rats with bilateral SBH (Sahu et al., 2019) show increased propensity to hyperthermia-induced seizures, and conducted histopathological examination to correlate seizure parameters with anatomical findings.

## Materials and Methods

### Animals

Animal experiments were performed in agreement with European directive 2010/63/UE and received approval from the French Ministry for Research, after ethical evaluation by the Institutional Animal Care and Use Committee of Aix-Marseille University [protocol number: 2015040809544569_v2 (APAFIS#436)]. *Dcx*-KD rats with bilateral SBH (*n* = 11) and mismatch controls (*n* = 9) were generated using tripolar *in utero* electroporation as described (Sahu et al., 2019). Successfully electroporated rats were selected 1 day after birth (P1) based on GFP expression by using transcranial illumination with a 460–495 nm emitting light source and a 500–550 nm filter (BLS Ltd.).

### Hyperthermic Seizures

**FIGURE 1:**
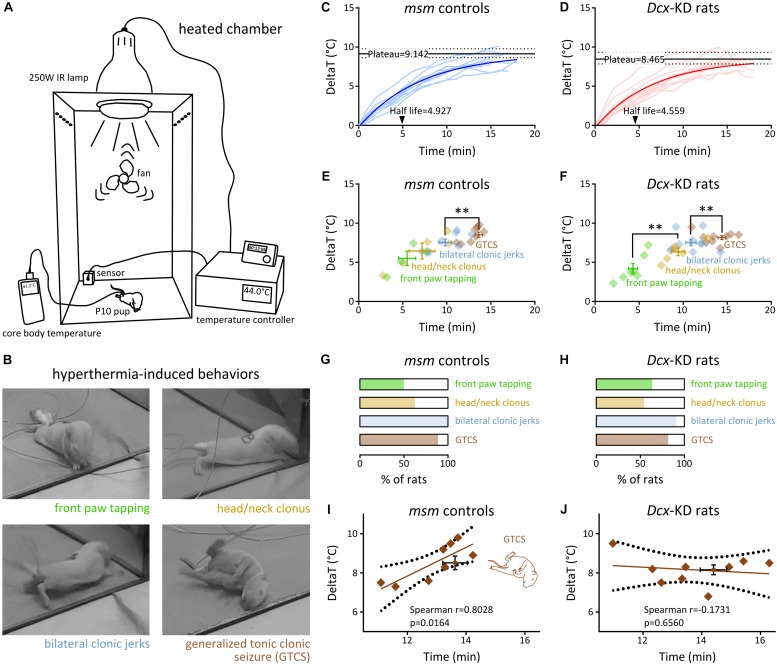
Hyperthermia-induced behavioral seizures in rats with bilateral SBH and controls. **(A)** Scheme of the experimental setup for induction of hyperthermic seizures in P10 rat pups using a heated chamber. **(B)** Snapshots of the four behavioral seizure manifestations induced by hyperthermia. **(C,D)** Line graphs illustrating hyperthermia-induced changes from baseline body temperature over time in mismatch controls **(C)** and *Dcx*-KD rats with bilateral SBH **(D)**. The light colored curves show changes in individual rats, and the dark colored curves show non-linear fits of the data with a one phase exponential decay. Solid black lines depict plateau temperatures and dashed lines show their 95% CI. Arrowheads indicate half-lifes. **(E,F)** Dot plots illustrating the temporal sequence of behavioral seizure manifestations culminating in generalized tonic clonic seizures, in relation with baseline temperature changes in mismatch controls **(E)** and *Dcx*-KD rats with bilateral SBH **(F)**. Crosshairs depict mean values and standard errors. Diamonds correspond to values for individual rats. **(G,H)** Stacked bar graphs illustrating the percentage of rats developing the different behavioral seizure manifestations in mismatch controls **(G)** and *Dcx*-KD rats with bilateral SBH **(H)**. **(I,J)** Scatterplots and best-fit linear regression lines illustrating the relationship between GTCS latencies and temperature thresholds in mismatch controls **(I)** and *Dcx*-KD rats with bilateral SBH **(J)**. Dotted lines depict 95% prediction bands of the regression lines. Crosshairs depict mean values and standard errors. ^∗∗^*p* < 0.01.

We utilized a modification of the hyperthermia-induced FS model (Holtzman et al., 1981; Schuchmann et al., 2006) where P10 rat pups are placed individually in a custom-made 30 × 30 × 60 cm Plexiglass chamber (Figure 1A). An ambient temperature of 44°C was maintained in the chamber using a 250 W infrared lamp, a temperature controller (Physitemp), and a thermocouple. The lamp was positioned 55 cm above the rat, and a small fan enabled air circulation. Body temperature was continuously monitored with a flexible rectal probe. Animal behavior was video-recorded for later analysis and scoring of seizure parameters. Experimenters were blind to the animals’ status.

### Histology

Less than 5 min after generalized convulsions have occurred, animals were deeply anesthetized with dolethal and perfused transcardially with 4% paraformaldehyde. Serial frontal sections (100 μm) were performed using a vibrating microtome (Leica Biosystems), mounted on glass slides, and images were taken using a fluorescence stereomicroscope (Olympus). Measurements were performed using Fiji (Schindelin et al., 2012).

### Data Analysis

Statistical analyses were performed using Prism 6 (Graph-Pad Software). Normality of the data distributions was tested using d’Agostino and Pearson test and Shapiro–Wilk normality test. All values are given as mean ± SEM. All tests were two-tailed, and significance level was set at *P* < 0.05.

## Results

To test whether rats with SBH were more prone to develop HS than controls, we induced hyperthermia using a heated chamber (the section “Materials and Methods” and Figure 1A) in 11 *Dcx*-KD rats with bilateral SBH, and 9 mismatch controls at postnatal day 10. This age was selected to model the period when brain development in rats is considered comparable to that of human infants at risk for FS (McCaughran and Schechter, 1982; Hjeresen and Diaz, 1988). In all the pups, hyperthermia induced behavioral changes, starting with rapid and apparently random movements described as hyperkinesia (McCaughran and Schechter, 1982; Baram et al., 1997), and followed by a period of apparent hypotonia or ataxia. Behavioral seizures with four main stereotyped manifestations were then observed (Figure 1B): (1) synchronous “tapping-like” movements of front paws (referred to as front paw tapping), (2) clonic movements or tremors of head and neck (referred to as head/neck clonus), (3) bilateral myoclonic limb jerks (referred to as bilateral clonic jerks), and (4) generalized tonic–clonic movements with loss of postural control (and apparent unconsciousness), and frequently accompanied by salivation and urination (referred to as generalized tonic–clonic seizure, GTCS). We considered these stereotyped manifestations as different stages of seizure severity, as described earlier (Hjeresen and Diaz, 1988), and scored them with respect to their latencies and temperature thresholds.

Baseline body temperatures prior to hyperthermia were not different (*P* = 0.8302) in mismatch controls (34.97 ± 0.28, *n* = 9) and *Dcx*-KD rats with bilateral SBH (34.88 ± 0.26, *n* = 11). We plotted the hyperthermia-induced changes from baseline body temperature (Figures 1C,D), and performed a non-linear fit with a one phase exponential decay. We found that the plateau temperature in *Dcx*-KD rats [8.465 ± 0.353, 95% confidence interval (CI): (7.847–9.324)] was lower than in mismatch controls [9.142 ± 0.275, 95% CI: (8.639–9.784)], and the half-life, shorter [4.559, 95% CI: (3.657–5.889) in *Dcx*-KD rats, and 4.927, 95% CI: (4.216–5.864) in mismatch controls].

Different behavioral seizure manifestations were found to occur sequentially in both groups of hyperthermia-exposed rats (Figures 1E,F). The order of occurrence of different manifestations was not different in mismatch controls and *Dcx*-KD rats. On average, front paw tapping was the first manifestation to occur, and was followed by head/neck clonus and bilateral clonic jerks. GTCS was the final seizure manifestation observed. Progression in this sequence of behavioral changes finally culminating in GTCS showed slight differences between groups: in *Dcx*-KD rats, front paw tapping occurred with significantly shorter latencies than head/neck clonus (4.31 ± 0.51 min and 9.44 ± 0.75 min, respectively; *p* = 0.0012), while this feature is not seen in mismatch controls (Figures 1E,F). In *Dcx*-KD rats, bilateral clonic jerks occurred slightly later than head/neck clonus (10.89 ± 0.58 min), and with significantly shorter latencies than GTCS (13.73 ± 0.55 min; *p* = 0.0041). A similar feature was observed in mismatch controls (9.82 ± 0.72 min for bilateral clonic jerks and 12.92 ± 0.37 min for GTCS; *p* = 0.0030). In addition to their sequential order of occurrence, different seizure manifestations were found to occur at increasing threshold temperatures, with the earliest manifestations developing at lower temperature changes from baseline (Figures 1E,F).

The proportions of rats developing different seizure manifestations in both groups were not different (Figures 1G,H). Front paw tapping was observed in 50% of mismatch controls and in 63.64% of *Dcx*-KD rats (4/8 and 7/11, respectively; *p* = 0.6577). Head/neck clonus was observed in 62.5% of mismatch controls and in 54.55% of *Dcx*-KD rats (5/8 and 6/11, respectively; *p* > 0.9999). Bilateral clonic jerks were found in 100% of mismatch controls and in 90.91% of *Dcx*-KD rats (8/8 and 10/11, respectively; *p* > 0.9999). GTCS developed in 88.89% of mismatch controls and in 81.82% of *Dcx*-KD rats (8/9 and 9/11, respectively; *p* > 0.9999).

Because we observed that seizure manifestations occurred sequentially and at increasing temperature thresholds, we plotted the latencies to GTCS against their temperature thresholds in both rat groups, and computed a linear regression analysis (Figures 1I,J). As expected, we detected a significant positive linear relationship between GTCS latencies and temperature thresholds in mismatch rats [Spearman *r* = 0.8028, 95% CI: (0.226, 0.9628), eight pairs of variables analyzed, *p* = 0.0164; Figure 1I]. Surprisingly, however, the same analysis in *Dcx*-KD rats revealed no relationship [Spearman *r* = −0.1731, 95% CI: (−0.7509, 0.5548), nine pairs of variables analyzed, *p* = 0.6560; Figure 1J], suggesting the presence of an additional, uncontrolled variable in the *Dcx*-KD group. In our previous report (Sahu et al., 2019), we have observed that adult *Dcx*-KD rats presented with bilateral SBH of variable positions and sizes. This prompted us to test whether inter-individual anatomical differences in *Dcx*-KD rats may be responsible for the disrupted linear relationship between GTCS latencies and temperature thresholds compared to mismatch controls.

**FIGURE 2:**
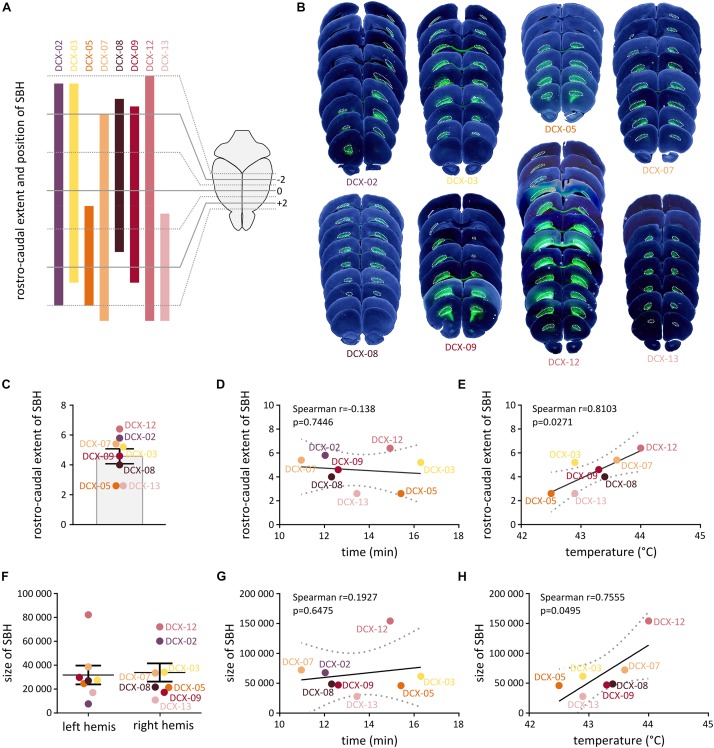
Histopathological characteristics of *Dcx*-KD rats with bilateral SBH. **(A)** Clustered bar graphs showing the rostrocaudal extent and position of SBH relative to Bregma in eight hyperthermia-exposed *Dcx*-KD rats. The rostrocaudal extent encompasses the distance between the most rostral end and the most caudal end of SBH in any of the two hemispheres. Animal identifier codes are given above each bar. **(B)** Composite images of bright-field and fluorescent microphotographs of serial neocortical sections from the eight hyperthermia-exposed *Dcx*-KD rats. Bilateral subcortical band heterotopia, mostly composed of green fluorescent protein (GFP)-expressing neurons are delineated with dotted lines. **(C)** Bar graphs showing the rostrocaudal extent of SBH in eight hyperthermia-exposed *Dcx*-KD rats. **(D,E,G,H)** Scatterplots and best-fit linear regression lines illustrating the positive linear relationship between the temperature thresholds and rostrocaudal extent **(E)** or size of SBH **(H)**, and the absence of relationship between GTCS latencies and rostrocaudal extent **(D)** or size of SBH **(G)**. Dotted lines depict 95% prediction bands of the regression lines. **(F)** Dot plots showing the size of SBH (depicted as cumulative area) in the left (left hemis) and right (right hemis) hemispheres of eight hyperthermia-exposed *Dcx*-KD rats.

We thus carried out systematic measurements of the size and anteroposterior extent of SBH from serial frontal brain sections of eight *Dcx*-KD rats exposed to hyperthermia-induced FS. SBH were found in all the rats, and they extended bilaterally with variable rostro-caudal distances and sizes (Figures 2A,B). In most cases, SBH were located in the white matter below the dorsal portion of the neocortex and extended over 4.57 ± 0.50 mm (Figure 2C). Their size, estimated from serial sections as cumulative areas, ranged from 31.82 ± 7.89 pixels in the left hemisphere to 33.89 ± 7.63 pixels in the right hemisphere (Figure 2F). Although the overall extent and position of SBH appeared roughly homogeneous at the population level, inter-individual anatomical differences were clearly visible. To detect potential anatomical relationships with the GTCS latencies and temperature thresholds in *Dcx*-KD rats, we thus plotted the latencies and temperature thresholds against the rostro-caudal extent (Figures 2D,E) and size (Figures 2G,H) of SBH, and conducted linear regression analyses. We detected a significant positive linear relationship between temperature thresholds and rostro-caudal extent of SBH [Spearman *r* = 0.8103, 95% CI: (0.1469, 0.9709), *p* = 0.0271; Figure 2E], as well as a positive linear relationship between temperature thresholds and size of SBH [Spearman *r* = 0.7555, 95% CI: (0.0057, 0.9615), *p* = 0.0495; Figure 2H]. However, no relationship was found when the latencies were plotted against the rostro-caudal extent (Spearman *r* = −0.138, *p* = 0.7446, Figure 2D) or the size (Spearman *r* = 0.1927, *p* = 0.6475, Figure 2G) of SBH. In all, these observations indicate that brains with SBH display variable degree of susceptibility to HS depending on the size and rostro-caudal extent of SBH, and that these two parameters do influence threshold temperatures but not seizure latencies.

## Discussion

Here, we report that when rats with bilateral SBH are subjected to hyperthermia, seizures are elicited with a lower plateau temperature and a shorter half-life compared to mismatch controls with no malformation. In both groups, hyperthermia induces a sequence of behavioral changes finally culminating in GTCS; however, progression in this sequence shows slight differences between groups. Also, while we could observe a linear relationship between the latencies of HS and their temperature threshold in control animals, this relationship is lost in rats with bilateral SBH, indicating variable degrees of susceptibility to HS. Histopathological examination shows that the variable degrees of susceptibility are due to inter-individual anatomical differences in size and extent of SBH, exerting influences over threshold temperatures but not over seizure latencies. In all, our observations suggest that a preexisting developmental defect such as SBH may predispose immature brains to FS and/or affect temperature thresholds for seizures. Further, this indicates that an association exists between the overall extent or location of a cortical malformation, and the predisposition to FS.

Patients with SBH typically present with epilepsy (85–96% depending on patient cohorts) and have their first seizure in the first decade (D’Agostino et al., 2002; Bahi-Buisson et al., 2013), a period in which children are susceptible to FS [from 6 months to 6 years (Verity et al., 1985a, b; Berg et al., 1992)]. Seizure types found at epilepsy onset often evolve, and a combination of multiple seizure types could be observed as patients grew older. Interestingly, Barkovich et al. (1994) reported the case of a female patient with a single febrile seizure at age 13 months, who went on to develop partial seizures with generalization 5 months later. Bahi-Buisson et al. (2013) described two cases of children with *DCX* mutations and a history of febrile seizure. One patient had FS at age 0.9 year and later developed complex partial seizures. The other had FS at age 2 years and was described as seizure-free on antiepileptic drugs 4 years later. Because these patients developed epilepsy several months after their FS, one could speculate that FS in these patients have contributed to the epileptogenic process, acting as a “second-hit” precipitating factor. Alternatively, it may simply reflect the fact that immature brains with preexisting developmental defects are more vulnerable to FS.

In the MAM rodent model of cortical dysplasia, prolonged hyperthermia-induced seizures at juvenile ages were found associated with a higher risk of developing spontaneous recurrent seizures at adult ages (Park et al., 2010). Similarly, in the freeze-lesion rodent model of focal cortical microgyri, atypical FS induced by hyperthermia at juvenile ages were found to result in spontaneous recurrent seizures in adults (Scantlebury et al., 2005). These rodent studies tend to suggest that FS in immature brains with preexisting malformations may act as a precipitating factor for epileptogenesis. Of interest, depending on the model used for inducing FS and on their duration, interictal abnormalities alone (Scantlebury et al., 2005) or associated with spontaneous recurrent seizures (Dube et al., 2006; Park et al., 2010) were also observed at adult ages in the absence of underlying symptomatic cause. Severities of spontaneous adult seizures, however, were lower than those of animals with cortical dysplasia or focal microgyri, suggesting that FS alone may not be sufficient. Because our *Dcx*-KD rats with bilateral SBH develop spontaneous seizures at age 2 months (Sahu et al., 2019), future work may help clarifying whether epilepsy onset could be shifted toward earlier ages when rats are exposed to hyperthermia-induced seizures. In addition, it would be important to assess whether other early-life insults besides hyperthermia could precipitate epileptogenesis in rats with bilateral SBH. Interestingly, rat pups exposed to early life stress due to maternal separation were found to exhibit a higher vulnerability to epileptogenesis in adult life (Salzberg et al., 2007; Kumar et al., 2011). In line with this, a rise in plasma corticosterone levels was reported in the freeze-lesion model of focal cortical microgyri, associated with lower thresholds for HS and a higher long-term vulnerability to develop epilepsy, especially in males (Desgent et al., 2012). In all, this suggests that in immature brains with preexisting developmental alterations, several types of early-life insults may act as precipitating factors for epileptogenesis, alone or in combination, in a “multiple-hit” fashion.

Previous works in rodent models have not assessed the impact of the anatomical extent of preexisting malformations on the predisposition to HS. This was mostly due to the distinct purpose of earlier studies, aiming at investigating the long-term consequences of HS and subsequent epileptogenesis. Here, our observations indicate that brains with SBH display variable degree of susceptibility to HS depending on the size and rostro-caudal extent of SBH. Of interest, larger SBH are associated with higher temperature thresholds, whereas smaller SBH show lower temperature thresholds. Interestingly, in the reported cases of SBH with a history of febrile seizure (Barkovich et al., 1994; Bahi-Buisson et al., 2013), two out of three patients had less severe forms of SBH with SBH grade 2 (out of 4). The reasons for this are unclear, but we have recently showed that distinct circuit-level defects can be found in the overlying cortex of *Dcx*-KD rats depending on the precise location and size of SBH (Plantier et al., 2018). Future work is needed to clarify whether distinct network changes related to the precise location and size of SBH could contribute to the variable degrees of susceptibility to HS in rats with SBH. Whether other contributing factors such as hyperthermia-induced respiratory alkalosis (Schuchmann et al., 2008), or altered regulation of body temperature remains an open question.

## Conclusion

In conclusion, we have shown that rats with bilateral SBH exhibit variable degrees of susceptibility to hyperthermia-induced seizure depending on their inter-individual anatomical characteristics. This is in line with the common view that immature brains with preexisting developmental alterations are more vulnerable to FS, and suggests that various predisposing factors and underlying symptomatic causes may contribute to the etiology of complex FS.

## Data Availability Statement

The datasets generated for this study are available on request to the corresponding author.

## Ethics Statement

Animal experiments were performed in agreement with European directive 2010/63/UE and received approval from the French Ministry for Research, after ethical evaluation by the Institutional Animal Care and Use Committee of Aix-Marseille University [protocol number: 2015040809544569_v2 (APAFIS#436)].

## Author Contributions

KM and J-BM carried out hyperthermia-induced seizures experiments and analyzed the data. KM performed the histological analysis. EB performed tripolar in utero electroporation. RM built the Plexiglass chamber. FW and AR contributed to the study design. J-BM conceived the study, made the figures, and wrote the manuscript.

## Conflict of Interest

The authors declare that the research was conducted in the absence of any commercial or financial relationships that could be construed as a potential conflict of interest.
